# Emergence of highly pathogenic avian influenza viruses H5N1 and H5N5 in white-tailed eagles, 2021–2023

**DOI:** 10.1099/jgv.0.002035

**Published:** 2024-11-01

**Authors:** Cathrine Arnason Bøe, Eve Marie Louise Zeyl Fiskebeck, Malin Rokseth Reiten, Johan Åkerstedt, Maryam Saghafian, Ragnhild Tønnessen, Britt Gjerset, Kjersti Sturød, Torfinn Moldal, Grim Rømo, Morten Helberg, Duncan Halley, Lars-Erik Lundblad Rondestveit, Knut Madslien, Silje Granstad

**Affiliations:** 1 Norwegian Veterinary Institute, P.O. Box 64, NO-1432 Ås, Norway; 2 BirdLife Norway, Sandgata 30 B, NO-7012 Trondheim, Norway; 3 Norwegian Institute for Nature Research, Høgskoleringen 9, NO-7034 Trondheim, Norway; 4 Norwegian Food Safety Authority, P.O. Box 383, NO-2381 Brumunddal, Norway

**Keywords:** H5N1, H5N5, *Haliaeetus albicilla*, histopathology, HPAI, surveillance

## Abstract

Highly pathogenic avian influenza (HPAI) poses a substantial threat to several raptors. Between 2021 and 2023, HPAI viruses (HPAIVs) of the Goose/Guangdong lineage H5 clade 2.3.4.4b became widespread in wild birds in Norway, and H5N1 and H5N5 viruses were detected in 31 white-tailed eagles (*Haliaeetus albicilla*, WTEs). Post-mortem examinations of four WTEs revealed no macroscopic pathological findings. Microscopic examinations showed the presence of myocardial and splenic necroses and a few lesions in the brain. *In situ* hybridization revealed the presence of the virus in several organs, suggesting a multisystemic infection. The detection of HPAIV H5N5 in a WTE in February 2022 marked the first recorded occurrence of this subtype in Norway. Since then, the virus has persisted, sporadically being detected in WTEs and other wild bird species. Phylogenetic analyses reveal that at least two distinct incursions of HPAIV H5N1 Eurasian (EA) genotype C affected WTEs, likely introduced by migratory birds from Eurasia and seabirds entering from Western and Central Europe. Some WTE isolates from 2021 to 2022 clustered with those from Canada and Ireland, aligning with the transatlantic spread of H5N1. Others were related to the 2021 mass mortality of great skuas in the UK or outbreaks in seabird populations, including gannets, gulls and terns, during 2022 in the North Sea region. This suggests that the WTEs were likely preying on the affected birds. Our study highlights that WTEs can act as sentinels for some HPAIV strains, but the absence of several known circulating genotypes in WTEs suggests varying pathogenic effects on this species.

## Data Summary

Supplementary material is available with the online version of this article, available through Figshare at 10.6084/m9.figshare.27089602 [1]*.* External sequence data for the haemagglutinin and neuraminidase segments of H5N1 and H5N5 influenza A strains collected from birds during the period between 1 January 2020 and 31 December 2022, and sequences used as outgroup (EPI_ISL_1254 and EPI_ISL_177506) to root phylogenetic trees were downloaded from the GISAID EpiFlu database (https://gisaid.org/) [2]. The sequence data generated in this study are available in the GISAID EpiFlu database. The reference data set used for genotyping was obtained from the European Union Reference Laboratory for Avian Influenza, also described by Fusaro *et al*. [3]. The detailed data included in each phylogenetic analysis and comprehensive descriptions of data pre-processing and analysis are provided in the Supplementary Material. This includes URLs and references to the software used. Scripts developed for visualization and annotation of phylogenetic trees are available in Zenodo (https://zenodo.org/doi/10.5281/zenodo.11143879), which corresponds to the release tag of the GitHub repository (https://github.com/NorwegianVeterinaryInstitute/WTE_AI_article).https://github.com/NorwegianVeterinaryInstitute/WTE_AI_article). In this repository, we have also included the logs that contain the commands used to pre-process the data when relevant.https://figshare.com/s/42571f2e8b1cc9d29738

## Introduction

Highly pathogenic avian influenza (HPAI) viruses (HPAIVs) of the A/Goose/Guangdong/1/96 lineage have been a recurrent concern in Europe for more than 20 years due to their rapid spread and high mortality among both poultry and wild birds [4,5]. In recent years, HPAI H5 clade 2.3.4.4b viruses have rapidly extended their geographical range, causing devastating outbreaks in wild bird populations [6,9]. Apex predators, such as raptors that hunt weakened prey or scavenge on infected birds, are at high risk of contracting HPAIV. Neurological disease and mortality have been observed in many raptor species worldwide following outbreaks caused by HPAI clade 2.3.4.4 viruses [10,16].

Wild aquatic birds of the orders *Anseriformes* and *Charadriiformes* serve as the natural reservoir of influenza A viruses (subtypes H1–H16, N1–N9) [17,18]. Most of these viruses are low pathogenic avian influenza viruses (LPAIVs), typically causing subclinical infections in these hosts [18,19]. However, HPAI H5 2.3.4.4b viruses can cause high mortality. There is significant genetic diversity among avian influenza viruses (AIVs) in nature, and the cocirculation of LPAIVs and HPAIVs creates opportunities for reassortment and the emergence of new virus strains [18]. The risk of such reassortment in wild birds increases when virus transmission is widespread, typically when birds gather in large numbers at breeding areas or feeding sites [20,21]. Reassortment may also occur in animal species susceptible to both avian and mammalian influenza A viruses [18]. In addition, the genome of AIVs consists of RNA, which is prone to genetic drift. The rapid genetic changes in circulating AIVs, particularly in the haemagglutinin (HA) and neuraminidase (NA) genes, which affect virulence and host specificity [18,19], have led the European Union Reference Laboratory (EURL) for Avian influenza and Newcastle disease to classify HPAIV strains detected in Europe since 2020 into genotypes based on their segment reassortment patterns [3].

In November 2020, HPAIV was detected for the first time in Norway [22]. During the 2020–2021 epizootic season (October 2020 to September 2021), the HPAI H5N8 clade 2.3.4.4b virus predominated, mainly affecting waterfowl and gulls. In 2021, the HPAI H5N1 clade 2.3.4.4b virus was introduced to Norway, leading to increased numbers of virus detections in wild birds and the first outbreaks in commercial poultry farms. This prompted intensified communication efforts and raised awareness. Ornithologists, outdoor enthusiasts and hunters were encouraged to actively report diseased or dead wild birds. As a result, the number of wild birds tested for avian influenza in Norway doubled from 2020 to 2021, according to the records from the Norwegian Veterinary Institute (NVI) [23,24].

The white-tailed eagle (*Haliaeetus albicilla*, WTE), also referred to as the sea eagle, is one of the largest birds of prey in Europe. They are opportunistic scavengers and predators that prefer vulnerable prey that is easy to capture. WTEs are primarily found in coastal regions, islands and large inland water bodies across Europe, with a particularly strong presence in Northern Europe. They prefer habitats offering a combination of open water for fishing, abundant prey and suitable nesting sites, such as cliffs and large trees, but can also breed on the ground [25]. In Norway, WTEs nest along the coastline from Agder in the south to Finnmark in the north, but are most abundant in the northern part of the country. Adult breeding birds are mainly stationary, while juveniles and sub-adults typically display a more nomadic behaviour [26]. In Norway, the species was categorized as strictly protected from 1968 [27]. By the 1970s, about 400 breeding pairs remained [25]. Stringent conservation measures were therefore enacted, including protection from hunting, poisoning and nest plundering, alongside initiatives for habitat restoration and supplementary feeding programmes [28]. As a result, the number of WTE breeding pairs in Norway increased and ranged from 2800 to 4200 by 2015 [29]. This represents a substantial portion of the European WTE population estimated at 10 400–14 600 breeding pairs [30]. The European and Norwegian WTE populations were assessed and listed as Least Concern on the red lists of the International Union for Conservation of Nature and the Norwegian Biodiversity Information Centre in 2020 and 2021, respectively [31,32]. The conservation success allows Norway to export juvenile WTEs to other countries to support reintroduction programmes [33,34].

While the efforts to conserve the WTE population in Norway have shown progress, the introduction of the HPAIVs in wildlife poses a novel threat. Between 2021 and 2022, HPAIV H5N1 and H5N5 emerged in WTEs in Norway. Here we describe and compare the occurrence of different HPAIV genotypes in WTEs and other wild avian species from 2021 to 2023. We present clinical signs, pathological findings and virus distribution in various tissues of WTEs. Phylogenetic analyses of HPAIV isolates from WTEs and other bird species worldwide during the same period are used to assess the relatedness of these viruses and possible cross-species transmission routes.

## Methods

### Surveillance systems

The Norwegian Food Safety Authority (NFSA) and the NVI conduct surveillance of avian influenza in Norway. Wild bird species within *Anseriformes* (ducks, geese and swans) and *Charadriiformes* (gulls) at priority locations are targeted for active surveillance of avian influenza, in accordance with recommendations of Commission Delegated Regulation (EU) 2020/689 [35]. In active surveillance, birds are sampled as part of hunting or bird ringing. Other avian species, such as raptors, are primarily sampled through passive surveillance, meaning they are tested if they are observed with clinical signs of HPAI or found dead. Occasionally, live wild birds are tested for research or export purposes.

### Sample collection and virus detection

Sick or dead wild birds were reported to and sampled by inspectors from the NFSA as part of the national surveillance programme for avian influenza [36]. In addition, juvenile WTEs were sampled and tested for AIV prior to export as part of reintroduction programmes in Ireland and Spain in 2022 [33,34]. Swabs were collected from the trachea or oropharynx and cloaca and immediately placed in a virus transport medium (either Universal Transport Medium or Amies) and dispatched via overnight express parcel service to the NVI for analysis. NVI is the national reference laboratory for avian influenza. The samples were either processed shortly after reception or frozen at −70 °C until processing.

Nucleic acids were extracted on a MagNA Pure 24 or 96 (Roche). Real-time reverse transcription (RT)-PCR targeting the matrix (*M1*) gene of the influenza A virus was used to detect the virus, as described by Spackman *et al*. [37]. Influenza A positive samples were further characterized by subtype-specific real-time RT-PCRs for subtypes H5, N1 and N5 [38,40]. Pathogenicity was determined either by Sanger sequencing of the HA cleavage site or HP H5 real-time RT-PCR [41]. The standard operating procedures from the former and current EURL for Avian Influenza and Newcastle Disease, the Animal and Plant Health Agency and the Istituto Zooprofilattico Sperimentale delle Venezie were followed.

### Epidemiological data

Submitted samples, including samples of the national wild bird influenza surveillance programme, and their test results were registered in the NVI’s laboratory management system (PJS, ver. 21.1.25.0). R code was used to retrieve, clean, interpret, aggregate and merge data with geocoordinates [42]. The ggplot2 was used to produce figures and the tmap for mapping [43,44].

### Pathological examinations

The dead eagles that underwent necropsy had initially been frozen and later sent by overnight express parcel service to the NVI. Post-mortem examinations were conducted under the biosafety level 3 conditions. All included WTEs had been confirmed positive for HPAI by PCR as described above, prior to necropsy. The WTEs were examined following standard necropsy procedures, and the following tissues were collected for histopathological analysis: heart, liver, lung, kidney, spleen, small intestine, pancreas and brain. All tissues were fixed in 4% buffered formalin. Paraffin-embedded tissue sections were stained with haematoxylin and eosin and examined by standard light microscopy.

Paraffin-embedded tissue sections of the brain, heart, lung and liver were used to detect viral sequences *in situ* by RNAscope [45]. The probe was purchased from ACD Bio (2.5 LS prov V-influenza AH5N8 M2M1 C1, Cat. Nr.# 1048228-C1). The target of this probe is viral RNA encoding the M protein, which is conserved between all influenza A viruses. Using the target probe, the slides were stained with the RNAscope LS Red automated kit (ACD Bio, Cat. Nr.# 322750) on a Leica BOND RXm (Leica Biosystems).

### Whole-genome sequencing

All samples with Cq < 30 in the *M* gene real-time RT-PCR were subjected to cDNA synthesis as described by Zhou *et al*. [46] before whole-genome sequencing. The templates for cDNA synthesis were nucleic acids extracted as described for PCR above. Briefly, cDNA synthesis and subsequent PCR were performed using the SuperScript III Platinum One-Step Quantitative RT-PCR system (Thermo Fisher Scientific, Waltham, MA, USA) and primers (MBTUni-12-DEG 5′-GCGTGATCAGCRAAAGCAGG-3′ and MBTUni-13 5′-ACGCGTGATCAGTAGAAACAAGG-3′) amplifying all eight genomic AIV segments. The PCR products were analysed and quantified on a TapeStation 4200 (Agilent Technologies, Santa Clara, CA, USA). DNA libraries were prepared from 200 to 400 ng DNA using Illumina DNA Prep (Illumina, San Diego, CA, USA) and sequenced using Illumina NextSeq/MiSeq (Illumina).

### Assembly and genotyping

Consensus sequences were obtained via a reference-based mapping approach of the INSaFLU pipeline (https://insaflu.insa.pt) [47] using default settings. We employed the following sequences deposited in GISAID [2]: A/Red_fox/Norway/2022-07-1768-2T/2022 (EPI_ISL_18455203) segment 1, A/H5N1/A/Goose/Guangdong/1/96 (EPI_ISL_1254) segment 2–7 and A/H5N1/A/chicken/Egypt_A10351A_2014 (EPI_ISL_177506) segment 8, as mapping references for H5N1 samples. A/sea_eagle/Norway/2022-07-198_22VIR3866-2 (EPI_ISL_12754536) was used as a mapping reference for H5N5 samples. Genotyping was performed as described by Fusaro *et al.* [3] by cluster analysis in MEGA X (version 10.1.8, https://www.megasoftware.net/)) [48] of every segment to Eurasian (EA) reference sequences provided by the EURL.

### Phylogenetic analyses

Two H5N1 sequences were used as outgroups for the datasets, and all external sequences of H5N1 and H5N5 avian samples that had been collected during the period between 1 January 2020 and 31 December 2022 were downloaded from the GISAID EpiFlu database [2].

Details of data composition, quality insurance and processing are provided in the Supplementary Material. In short, datasets were prepared separately for H5N1 and H5N5 for the two segments: HA and NA.

A multiple sequence alignment (MSA) was produced for both segments and both subtypes (H5N1 and H5N5) separately. This was done using the Jalview (v2.11.2.6, https://www.jalview.org/) [49] server, with the muscle protein alignment algorithm, MUSCLE v3.8.31 [50], using pre-selected default parameters for H5N5 and a set of reference sequences for H5N1. The reference sequences MSA (H5N1) were then used as a guide to align H5N1 data with MAFFT (v7.520, https://mafft.cbrc.jp/alignment/software/)) [51,52]. Only sequences with a minimum length of 1700 bp for HA (*n* = 123) and 1400 bp for NA (*n* = 86) segments were kept in the MSA.

Our final datasets were composed of 84 and 82 sequences for H5N5 at HA and NA segments, respectively. The H5N1 dataset was composed of 4898 and 4744 sequences for HA and NA segments, respectively (see additional information in the Supplementary Material).

The maximum likelihood (ML) phylogenetic inference was performed identically on each dataset using the IQTREE (v2.2.9, http://www.iqtree.org/) COVID-edition for Linux 64-bit [53]. The ModelFinder [54] was used to automatically find the most appropriate evolutionary model for each dataset, which was then employed for phylogenetic inference. Branch support values were estimated using the ultrafast bootstrap [55,56].

Phylogenetic tree visualization was performed with R [57] custom scripts available in Zenodo (DOI: 10.5281). All represented phylogenetic trees are ML consensus trees that have been rooted using outgroup sequences posterior to phylogenetic inference.

## Results

### Detection of AIVs in WTEs

**Fig. 1.**
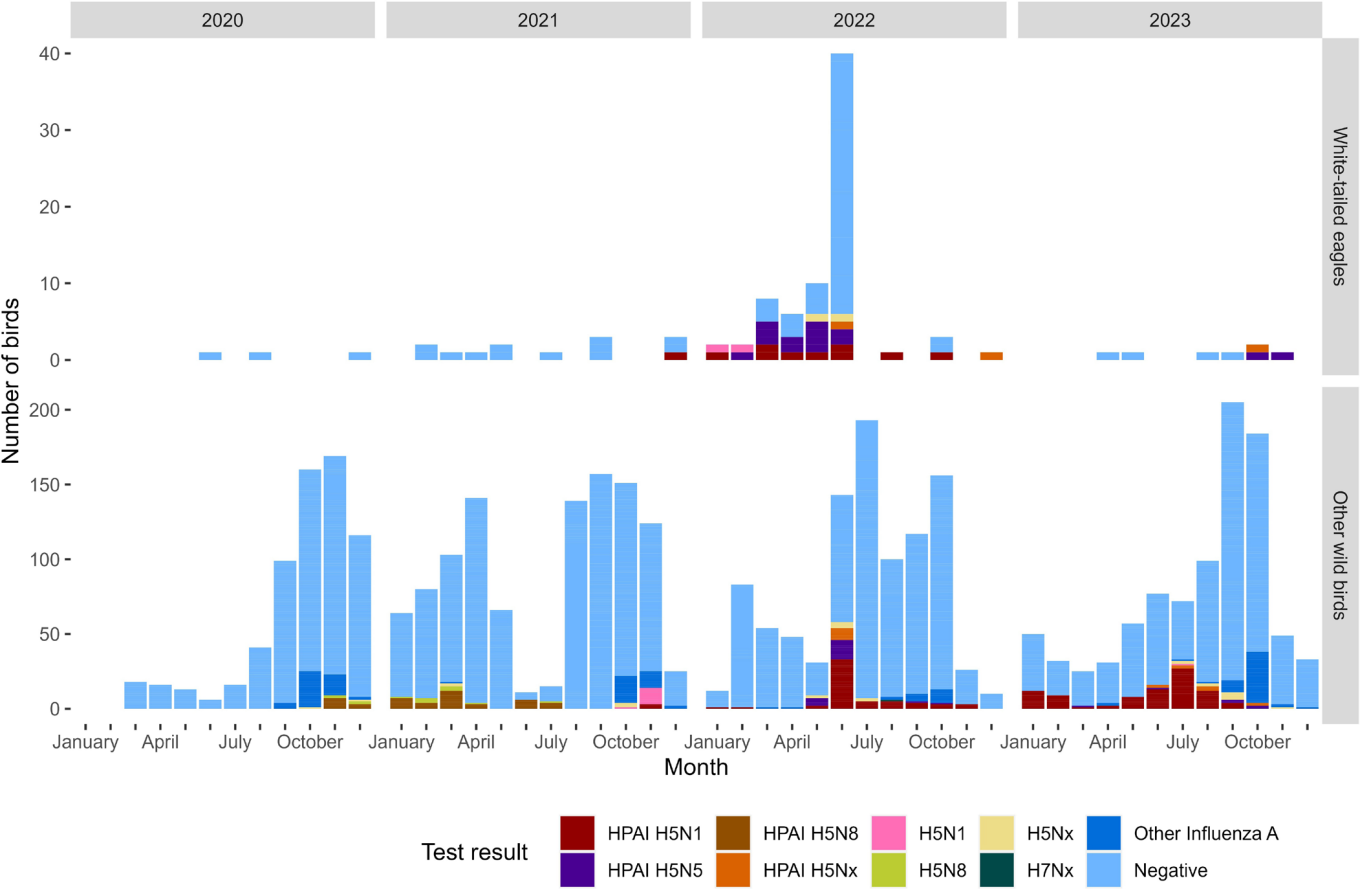
WTEs and other wild birds in Norway sampled and tested for AIV from January 2020 to December 2023. The results of subtyping and pathotyping are shown for the positive samples by colour codes in the square boxes. Negative samples are represented in light blue. Some samples had insufficient amounts of virus for complete NA-subtyping or pathotyping, and these are labelled Nx or lack the label HPAI, respectively.

From January 2020 to December 2023, 96 WTEs and 3648 other wild birds were tested for AIV through active and passive surveillance in Norway. Of these, 31 WTEs, all sampled as part of passive surveillance, tested positive between December 2021 and November 2023, as presented in Fig. 1 and Table S1, available in the online Supplementary Material of this article. AIV was not detected in WTEs during the 2020–2021 epizootic season (Fig. 1).

The majority of AIV-positive WTEs were detected throughout 2022 with three additional cases identified towards the end of 2023. A total of 706 other wild birds were tested for AIV through active and passive surveillance from December 2021 to December 2022 in Norway. Among these, HPAIV H5N1 was detected in 55 birds and H5N5 was detected in 15 birds (Table 1). H5N1 was predominantly detected in birds from *Sulidae* (*n* = 33) and *Laridae* (*n* = 14), while H5N5 was primarily found in birds from *Laridae* (*n* = 11) (Table 1).

**Table 1.** WTEs and other wild birds (grouped by family) positive for HPAIV H5N1 or H5N5 sampled between December 2021 and December 2022 in Norway

Birds	HPAI H5N1	HPAI H5N5	HPAI H5Nx
*H. albicilla*	10 (2)^†^	12	2 (2)^†^
*Sulidae*	33	1	7
*Anatidae*	4	-	-
*Laridae*	14	11	-
Other birds^*^	4	3	1
Total (birds other than WTE)	55	15	8

* These birds were from the families *Accipitridae* (*n* = 1), *Corvidae* (*n* = 3), C*olumbidae* (*n* = 1), *Procellariidae* (*n* = 1) and *Strigidae* (*n* = 1), in addition to one eagle of undetermined bird species.

† Isolates with incomplete pathotyping indicated in parenthesis for the WTEs.

**Fig. 2.**
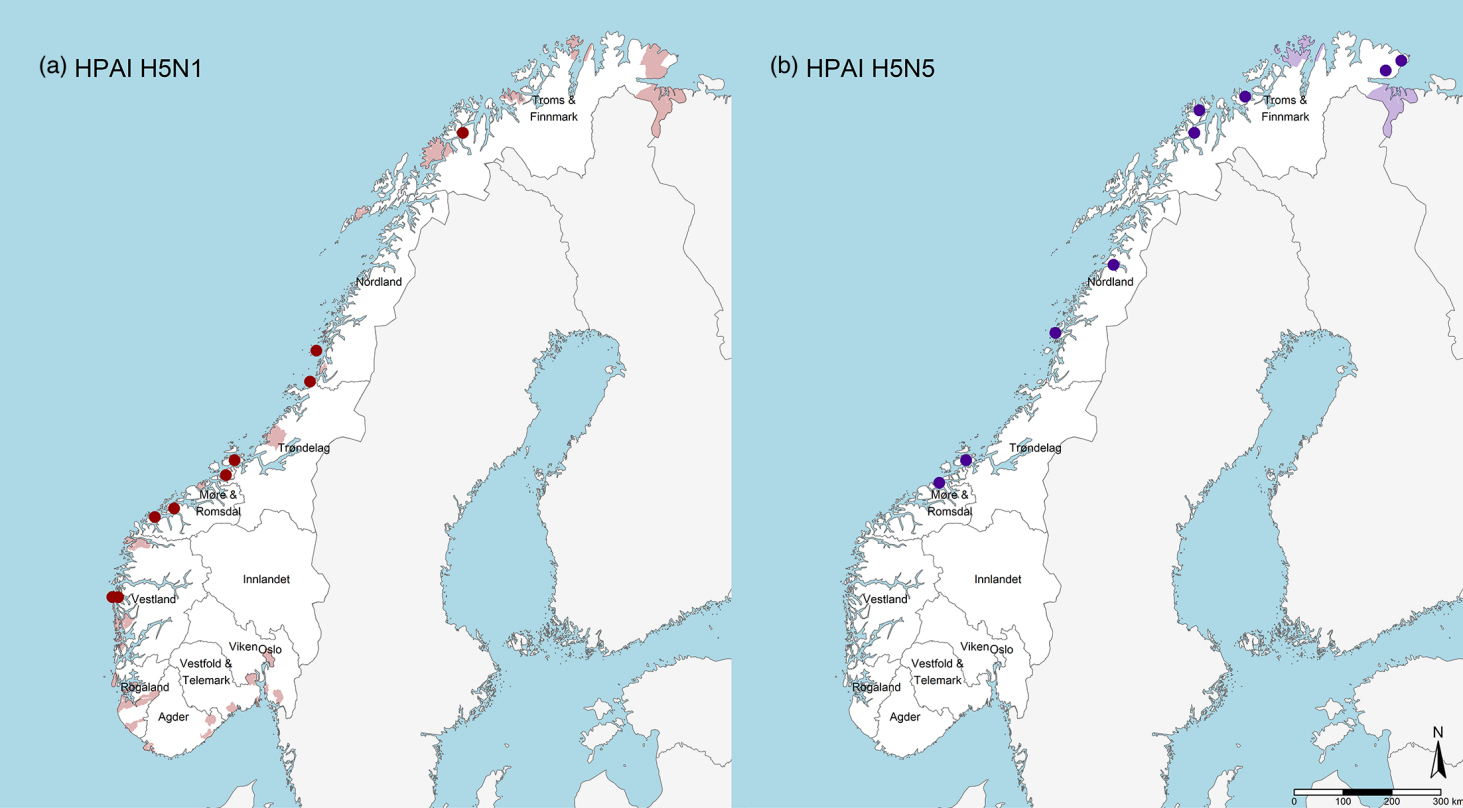
Detection of HPAIVs in WTEs and other wild bird species on mainland Norway from December 2021 to December 2022. (**a**) HPAIV H5N1 in WTEs (red dots) and municipalities where one or more other wild birds were tested positive for H5N1 (red patches). (b) HPAIV H5N5 in WTEs (purple dots) and municipalities where one or more other wild birds were tested positive for H5N5 (purple patches).

The HPAIV-positive WTEs were found on the west coast of Norway, extending to the northernmost region of the country (Fig. 2a, b). The other wild birds infected with HPAIV H5N1, mainly northern gannets (*Morus bassanus*) and various gulls, were found scattered along the Norwegian coastline (Fig. 2a). In contrast, on mainland Norway, H5N5 was exclusively detected in other wild bird species, primarily gulls, within the northernmost county of Finnmark (Fig. 2b).

### Antemortem observations and pathological examinations

**Fig. 3.**
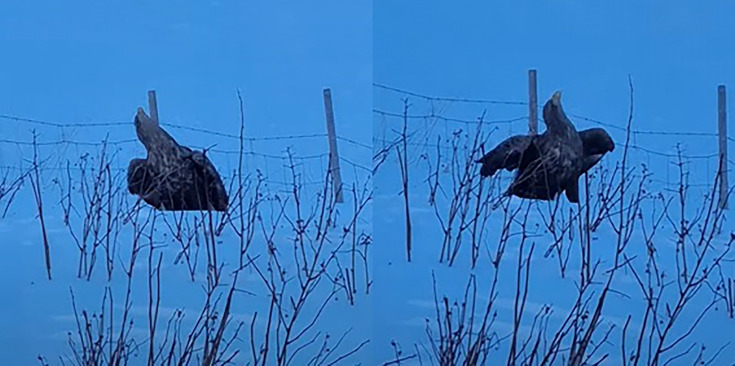
In early February 2022, an adult male WTE (no. 4, Table S1) was observed in northern Norway, exhibiting neurological signs such as torticollis, hyperextension of neck/opisthotonos and impaired flying ability. Infection with H5N1 was later confirmed by PCR. Sequencing of the H5 cleavage site to determine pathogenicity was not successful due to the low viral load in the samples. A video featuring the affected WTE can be accessed through the Supplementary Material on Figshare or the NVI’s YouTube account: https://youtu.be/bD0Kb-OUSk4. Photo: Jørn Johnsen.

HPAIV-infected WTEs (*n* = 4) encountered alive in the wild showed neurological signs such as torticollis, ataxia and impaired flight capabilities (Fig. 3 and video in the Supplementary Material). Three of these birds were euthanized by responsible authorities on welfare grounds due to severe illness. The fourth eagle died under observation.

Four WTEs received for post-mortem examination originated from three different counties in Norway (see Table S1 for WTE number): Troms (no. 4, Karlsøy), Nordland (no. 6, Kristiansund and no. 12, Herøy) and Møre and Romsdal (no. 8, Aure). HPAI H5N5 was confirmed in two of the WTEs (no. 6 and 12) and H5N1 in the other two (no. 4 and 8). All four eagles were adult males with normal body conditions (Table 2).

**Table 2.** Macroscopic and microscopic observations in WTEs subjected to necropsy

WTE no.*	Gender	HPAIV subtype	Observation	Macroscopic finding	Histological analysis	*In situ* hybridization
**4**	Male	H5N1	Observed alive exhibiting neurological symptoms and had difficulties flying; later found dead	Hyperaemic skin around eyes and beak; empty gizzard and intestines	Mild, multifocal lymphocytic and histiocytic myocarditis; multifocal splenic necrosis	Positive signal in splenic necroses; few neurons positive in the brain
**6**	Female	H5N5	Observed alive, exhibiting abnormal behaviour, not able to fly; euthanized	Weight 5.5 kg;sparse content in gizzard	Mild, multifocal lymphocytic and histiocytic myocarditis; multifocal splenic necrosis; mild encephalitis	Few neurons positive in the brain
**8**	Unknown	H5N1	Found dead	Weight 6.5 kg; dark red lungs; empty gizzard; sparse content in intestines	Multifocal to coalescing myocardial necrosis; mild encephalitis	Positive signal in necrosis, myocytes and vessels in the heart; strong positive signal in neurons and vessels in the brain
**12**	Female	H5N5	Found dead, with gunshot wounds	Haemorrhages and perforations in muscles related to trauma; empty gizzard	Mild, multifocal lymphocytic and histiocytic myocarditis; multifocal splenic necrosis; mild encephalitis	Analysis not performed

* The WTE no. relates to the numbering in Table S1.

The WTEs were received frozen and hence presented with varying degrees of freezing and thawing artefacts causing pink to red discolouration of adipose tissue and internal organs. Necropsies revealed non-specific pathological changes (Table 2). Two of the WTEs (no. 6 and 12) were euthanized by gunshot, resulting in perforations and haemorrhages in the body cavities that could mask other pathological changes present. A consistent finding in all birds was the lack of feed in the gizzard. WTE no. 8 exhibited dark red, congested lungs and fluid-filled air sacs, potentially related to freezing and thawing, and WTE no. 4 displayed red discolouration (hyperaemia or congestion) in the skin surrounding the eyes and beak.

Histological examination identified pathological changes in the heart of all four WTEs (Table 2). In three WTEs (no. 4, 6 and 12), there were multifocal myocardial accumulations of mononuclear inflammatory cells, mainly lymphocytes and histiocytic cells, either in connection to blood vessels or in the interstitium. Mild interstitial fibrosis surrounded the cell accumulations. In WTE no. 12, the changes were more widespread than in the other two. Multifocal to coalescing degeneration and necrosis of myocytes were observed in the myocardium of WTE no. 8. Few polymorphonuclear granulocytes (heterophiles) could be seen within these lesions in some areas. Multifocal splenic necroses of acute character and varying sizes were found in three WTEs (no. 4, 6 and 12). The assessment of the spleen from WTE no. 8 was difficult due to marked autolysis. Pancreatic tissues from three WTEs were examined (no. 4, 6 and 12), and no apparent pathological changes were detected apart from mild autolysis.

Examination of the brain was challenging due to autolysis and freezing and thawing artefacts. However, in brains from all WTEs, very mild perivascular infiltrations of mononuclear cells were suspected in some areas. Neurons in many areas were surrounded by round cells, either histiocytes or glial cells, possibly indicating neuronal degeneration or necrosis. The changes were the least expressed in the brain of WTE no. 4, and most marked in the brain of WTE no. 8. In the brain of no. 8, multifocal areas of cell infiltration and neuronal necrosis were seen in grey matter, in addition to mild perivascular cell infiltration. There were no evident pathological changes in other examined tissues.

**Fig. 4.**
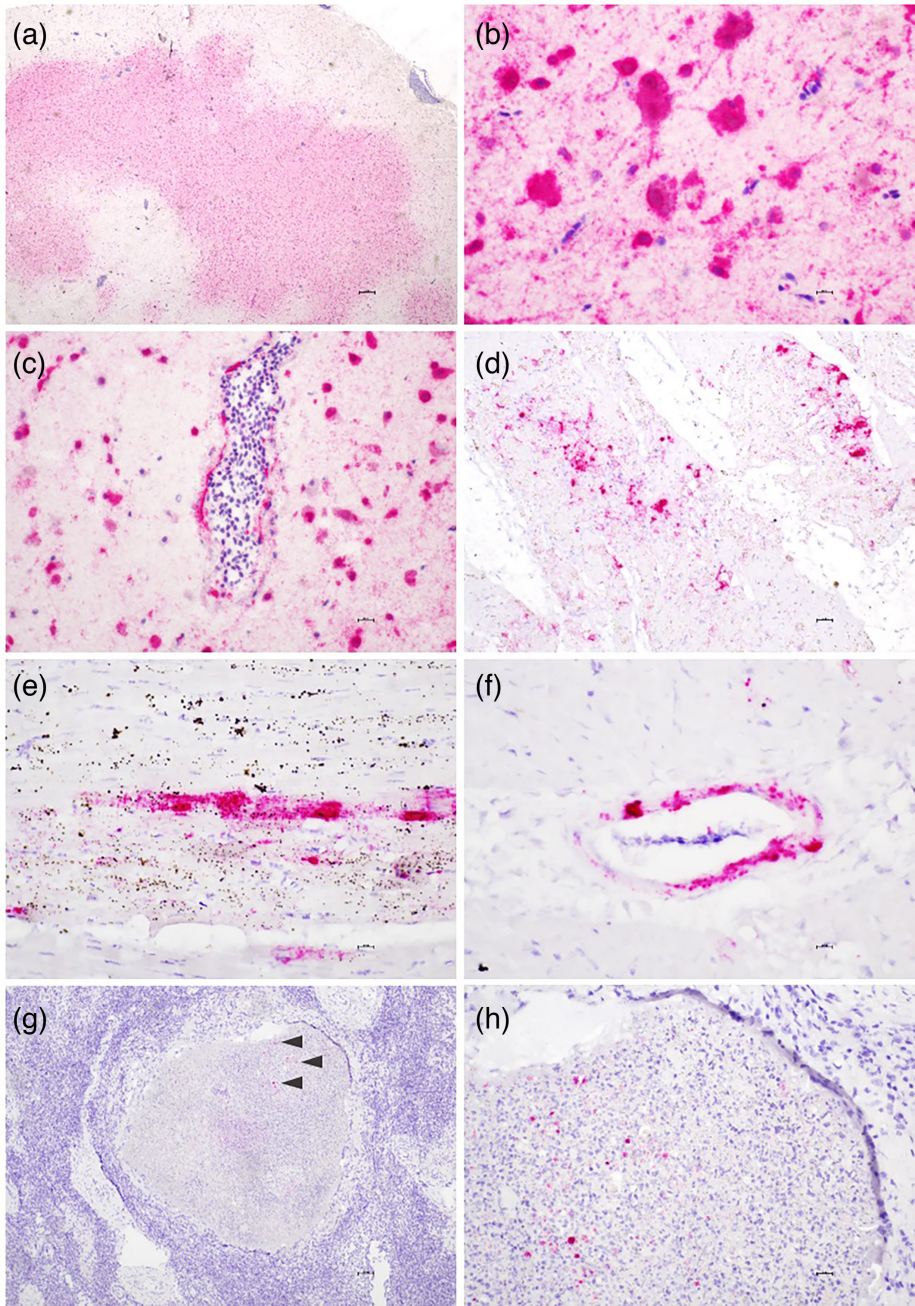
Microscopic findings by ISH of the brain, heart and spleen in WTEs infected with HPAIV. The presence of influenza A virus gene *M1* appears red. (a–c) Brain, eagle no. 8. (**a**) Widespread positive signal in neurons. (**b**) Positive signal in neurons and glial cells. (**c**) Viral RNA was detected in endothelial cells and the vessel wall of a venule. (d–**f**) Heart. (**d**) Scattered positive signals in myocytes colocalized to areas of myocyte degeneration and necrosis (haematoxylin and eosin stained slide not included). (**e**) Positive signal in heart myocytes. (**f**) Viral RNA was detected in the arteriolar wall. (**g–h**) Spleen. (**g**) Splenic necrosis with viral RNA in mononuclear inflammatory cells within the necrosis. (**h**) Scattered positive signal in mononuclear inflammatory cells within the necrosis.

Visualization of virus RNA by *in situ* hybridization (ISH) in selected tissues from WTEs no. 4, 6 and 8 revealed strong positive signals in several areas of the brain of WTE no. 8 (Fig. 4a–c). The staining was strong in neurons and extended into axons and dendrites. In some arterioles and venules in both the brain and heart, a positive signal was detected in endothelial cells lining the vessel lumens and the myoepithelium of the intimal wall. In brains (from WTEs no. 4 and 6), signals could be detected in spots in very few neurons. No signal could be detected in examined slides from other tissues from WTE no. 4 (lung, liver, kidney and intestine). Scattered positive signals were detected in the heart of one eagle (no. 8) (Fig. 4d–f). These signals were either associated with necrotic areas or could be seen in myocytes. Labelling of the mononuclear cell infiltrates was negative in all three WTEs. A scattered positive signal was detected in mononuclear inflammatory cells within areas of splenic necrosis in one WTE (no. 4) (Fig. 4g, h).

### Genetic analyses

**Fig. 5.**
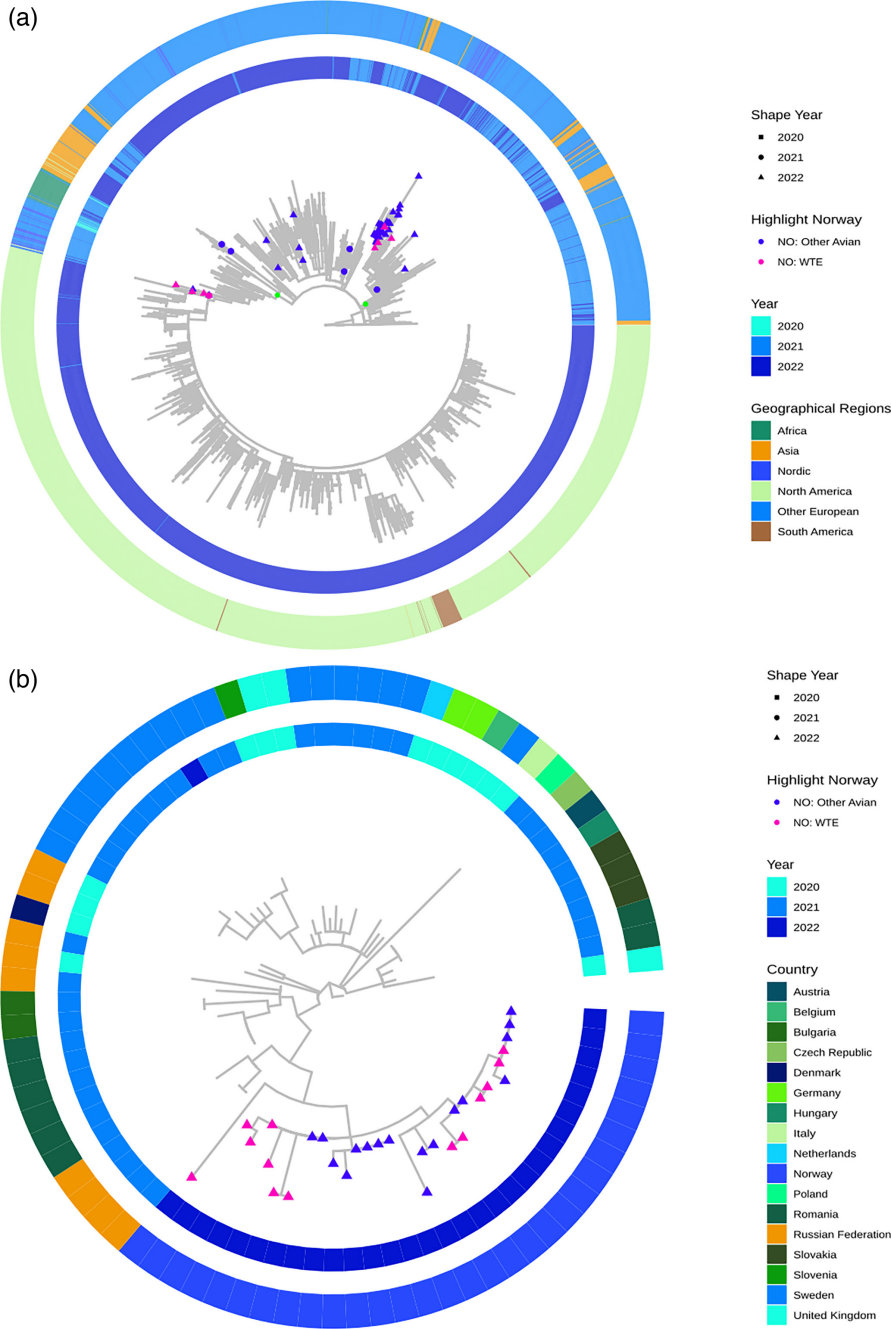
Consensus ML phylogenetic trees inferred from the HA gene segments of HPAI H5N1 (**a**) and H5N5 (**b**) viruses detected worldwide 2020−2022. The trees are rooted. The outgroups used for rooting have been pruned due to large distances to the rest of the tree bodies and are therefore not displayed. The light green dots in (**a**) represent the node of origin of the two groups presented as subtrees (Fig. S1d, f). Norwegian WTE isolates are highlighted in pink (two isolates appear as duplicates as they were also sequenced by the EURL and deposited to GISAID under different internal IDs: EPI_ISL_12754 536 and EPI_ISL_12754535). Isolates from other birds in Norway are highlighted in blue. The inner circle shows the year the isolates were obtained. The outer circle depicts the originating continent (**a**) or country (**b**) of the isolates. Nordic countries include Norway, Denmark, Finland, Iceland and Sweden. This figure can be viewed in greater detail on the Figshare link.

AIV genotypes EA-2021-I (H5N5) and EA-2020-C (H5N1) were identified in samples from the WTEs (Table S1). The HPAIVs from other wild birds also belonged to these genotypes, but in addition, seven isolates of genotype EA-2021-AB (H5N1) were detected (Table S2). The different EA genotypes will, hereafter, be referred to by their letter code for simplicity. To provide an overview of the WTE and other Norwegian wild bird isolates in relation to isolates detected worldwide from 2020 through 2022, phylogenetic analyses of the HA (Fig. 5a, b) and NA (Fig. S1) segments were carried out. Results regarding the evolutionary model used are provided in the Supplementary Material.

The phylogenetic analyses of the HA and NA segments both indicated that HPAIV isolates of subtype H5N1 from the Norwegian WTEs clustered into two distinct groups (Figs 5a and S1a). However, the branch support was insufficient to consider them as monophyletic clusters (see Figs S1b–f). Similarly, most Norwegian HPAIV H5N1 isolates from other wild birds also belong to these two groups. Seven isolates belonging to the genotype AB (Table S2) were associated with multiple sister groups. In Fig. S1b, c, showing collapsed rectangular plots of Figs 5a and S1a, the two groups containing HPAIV H5N1 isolates from WTE are pinpointed and, hereafter, named Node 9 (HA) and Node 15 (NA) (Fig. S1d, e) and Node 529 (HA) and Node 1732 (NA) (Fig. S1f, g).

Nodes 9 and 15 represent isolates that appear to have evolved from H5N1 viruses detected in Central and Eastern Europe and Russia. Four HPAIV WTE isolates (birds no. 14, 22, 26 and 27, Table S1) within these two nodes cluster with isolates from other Norwegian wild birds and isolates from countries around the North Sea (UK, Germany, the Netherlands and Belgium), as well as France, Spain and Sweden. Seabirds in *Laridae* and *Sulidae* were the most prominent within this cluster. Some spillover to or from poultry (*Gallus gallus* of the family *Phasianidae*) appears to have occurred in the UK, where several poultry isolates were almost identical (i.e. very low patristic distance) for both HA and NA segments to those found in wild birds (Fig. S1d, e, respectively). An additional isolate obtained from a WTE (bird no. 21, Table S1) also clustered within Nodes 9 and 15, but in a separate group, displaying high similarity to isolates obtained from birds of other families, specifically *Accipitridae* or *Anatidae,* in the UK.

In Fig. S1f, g (HA and NA, respectively), Nodes 529 and 1732 comprise several groups, which include isolates obtained in Norway. One monophyletic group of Asian isolates incorporated two Norwegian HPAIV isolates from ducks. Another monophyletic group contained four HPAIV isolates from WTEs (no. 1, 2, 8 and 11, Table S1), along with several sister subgroups. These subgroups encompassed isolates initially sampled in Africa and subsequently in Europe. Furthermore, some of these sister subgroups include isolates from birds collected in Central Europe and Russia through 2020 and 2021. Within this network, a significant monophyletic group emerged, stemming from one of these subgroups. This large cluster encompasses several sub-lineages derived from isolates collected in Europe between 2021 and 2022, with a high number of samples from 2021. One notable sub-lineage predominantly consists of isolates obtained from mostly poultry in North America, with additional representation from South America and Europe, including the four WTEs and other birds from Norway. This substantial monophyletic group primarily comprises isolates from 2022, except for European isolates, which appear to be a blend of samples collected in both 2021 and 2022. The Norwegian WTEs (no. 1, 2, 8 and 11, Table S1) in this group were sampled in late 2021 and early 2022, in contrast to the isolates residing within Node 9 and 15 that were all sampled from WTEs later in 2022. The European isolates of this cluster were derived from the ancestor of a group of isolates from birds belonging to *Accipitridae*, *Anatidae*, *Laridae* and *Stercorariidae*, collected across the British Isles, multiple Nordic countries, as well as other countries surrounding the North Sea, such as the Netherlands and Germany.

The Norwegian isolates of subtype H5N5, genotype I, originated solely from Europe and Russia for both segments (Figs 5b and S2a for HA and NA, respectively) within the investigated timeframe. Norwegian WTEs (birds no. 5, 9, 10, 12, 13, 15–18, 20 and 23, Table S1) and other Norwegian wild birds (mostly *Laridae*, one *Sulidae,* and two *Corvidae*), all sampled in 2022, along with Russian isolates from late 2021 (four for HA and two for NA segments), belong to a monophyletic cluster. The HA segment of bird no. 12 (EPI_ISL_18901607) was deposited to GISAID and included in the phylogenetic analysis, although the sequence was incomplete. This isolate thus appears as an outlier in the HA plots (Figs 5b and S2b). Some sequences from Norwegian wild birds and WTEs must have been identical (patristic distance is null), and the internal subgrouping of those isolates is not known (poor bootstrap support) both for HA and NA segments (rectangular plots of HA and NA shown in Fig. S2b, c, respectively). This, in addition to the generally short patristic distances between Norwegian birds and WTEs, indicates that those isolates are genetically homogenous. Moreover, both segments indicate that the Norwegian HPAIV H5N5 isolates are most closely related to isolates from Russia in 2021.

## Discussion

The phylogenetic results showed that the distributions of HPAIV isolates obtained from WTEs and other wild birds in Norway were partially overlapping. Raptors that frequently prey on infected carcasses could serve as sentinels for monitoring the presence of HPAIV in wild birds and other wildlife populations. However, HPAI surveillance in birds of prey is challenging in Norway. Due to legal protection, sampling mostly relies on passive surveillance of diseased and dead birds. In addition, Norway’s elongated geography and low human population density may reduce the likelihood of detecting such birds. Despite these limitations, our study demonstrates that monitoring HPAIV in WTEs provides some insights into the occurrence of the infection within a given region or country. Furthermore, it also sheds light on the genetic characteristics of HPAIVs circulating. These findings align with previous research, which revealed that both retrospective and prospective surveillance efforts identified avian raptors as significant indicators of HPAIV presence and genetic diversity [58]. Wild birds within *Accipitriformes* (hawks, eagles, vultures and kites) have been infected with HPAIVs during recent avian influenza epizootics in Europe [59,60]. On the contrary, neither LPAIVs nor HPAIVs were detected in a large-scale screening of raptors, including hundreds of WTEs and Peregrine Falcons (*Falco peregrinus*), during 2006–2007 in Sweden [61]. However, this study focused on nestlings, emphasizing that sampling sick or dead raptors may be a more efficient strategy for using raptors as sentinels for monitoring HPAIVs during avian influenza epizootics. A recent study by Hall *et al.* explored this by sampling raptors admitted to a rehabilitation centre in the USA and found the AIV genotypes in the raptors to be representative of virus genotypes found through surveillance of wild birds within the given time period [15]. Including sampling from wildlife rehabilitation centres could augment disease surveillance.

Our analyses across multiple epizootic seasons in Norway revealed a shifting landscape of detected HPAIV subtypes and genotypes. The first detection of HPAI clade 2.3.4.4b viruses in Norway was made in wild birds and belonged to subtype H5N8. This subtype has not been detected in WTEs in Norway. In 2021–2022, genotypes AB, C and I were found (this study). The following season, genotype EA-2022-BB emerged in Norway alongside I and AB [62,63]. Throughout 2022, genotypes C and I were identified among WTEs, but AB that circulated in other wild bird species in Norway during the same period has not been detected in WTEs in Norway (Table S2). In 2023, AB and BB were the predominant genotypes circulating in wild birds in Europe [64], but that year only genotype I was identified in Norwegian WTEs. The absence of several HPAIV subtypes and genotypes in WTEs known to circulate could be attributed to one or more of several reasons. It is possible that WTEs acquired immunity from previous AIV infections, or there may be host factors or virus characteristics that make WTEs less susceptible to certain genotypes. In 2023, WTEs were observed in regions experiencing significant mass mortality among black-legged kittiwakes (*Rissa tridactyla*) due to BB genotype (personal observation by Grim Rømo, 26 July 2023). Despite the likelihood of WTEs preying on infected kittiwakes, no apparent signs of morbidity or mortality were observed among the WTEs in the area. This observation raises questions about whether WTEs possessed immunity to BB, or alternatively, if they were less susceptible to this genotype. The BB genotype exhibits notable differences in segments 1, 3, 5, and 8 compared to I and C. Segments 3, 5 and 8 of BB are derived from H13 AIV, a subtype that primarily infects gulls and is considered gull-adapted [65,67]. This genotype may therefore be less adapted to replication in WTEs. The successive waves of different HPAIV genotypes sweeping across Europe have affected different groups of bird species during different epizootic seasons. This also underscores the complex interplay between viral genotypes and host factors for HPAIV transmission.

The lack of detections of HPAIV H5N8, as well as genotypes AB and BB, from WTEs in Norway could be explained by insufficient sampling. During the winter of 2016–2017, several WTEs died from HPAIV H5N8 infection in Germany [13], strongly arguing that WTEs are not immune to this subtype. In Norway, subtype H5N8 was the first HPAIV detected and affected mainly (*n* = 13) gulls, swans and ducks in the southern and western parts of Norway from November 2020to July 2021 [22]. Few detections in other bird species, and only at locations outside the main WTE-populated region, suggest a low probability of detecting H5N8 in WTEs.

Genotype AB has been detected in raptors in Europe through 2020–2022 [3]. We cannot rule out that there is variable susceptibility between different raptor species for genotype AB. However, the prevalence of HPAIV AB in other birds in Norway appears to have been low, with only seven detections from November 2021 to December 2022 (this study), indicating a low probability of detecting AB from WTEs. Genotype BB was not detected in raptors in Europe through the epizootic wave of 2021–2022, although it was found to a large extent in seabirds, which are part of their diet. This could indicate that raptors have an increased tolerance to this genotype, which may explain why no WTEs were found HPAIV-positive when genotype BB surged in Norway. It thus appears that a combination of low prevalence in other wild birds (H5N8 and genotype AB) and reduced susceptibility (genotype BB) can explain the lack of detections of these variants in WTEs.

### Several incursions of HPAIV into WTEs in Norway

In recent years, Europe has experienced multiple epizootic waves of different HPAI H5Nx clade 2.3.4.4b viruses in birds. The HPAI epizootic season of 2021–2022 marked the transition to an enzootic state in the European wild bird population [68]. Our results indicate that there were at least two incursions of HPAIV H5N1 genotype C into the WTE population in Norway. The HA lineage from which these isolates originate was split into two distinct branches at an earlier time point; one lineage persisted in Europe (represented in our Node 9), whilst the other lineage (represented in our Node 529) later spread over the Atlantic Ocean to America [68,69].

During the spring and summer of 2022, large outbreaks were observed in seabird populations, including gannets, gulls and terns, particularly in the North Sea region [70,71], and the incursions represented in Node 9 correlate with these. Specifically, two HPAIV isolates obtained from Norwegian WTEs during the spring of 2022 exhibit a close genetic relationship to isolates from wild birds in England (EPI_ISL_13370416, EPI_ISL_13370516 and EPI_ISL_13370895) sampled in the transition from 2021 to 2022 [69]. In late summer and autumn, two additional isolates from WTEs were obtained, clustering with isolates from northern gannets and gulls in the North Sea region, collected during the spring and summer of 2022.

The lineage represented in our Node 529 contains isolates constituting the first spread of HPAIV genotype C into WTEs in Norway and aligns with the timeline of numerous outbreaks in both wild birds and poultry across Europe during 2021–2022 [3,68]. One WTE isolate (EPI_ISL_18901647) and one great skua (*Stercorarius skua*) isolate from Svalbard (EPI_ISL_18901634) were highly related to two great skua isolates from the Western Isles, Scotland, UK. There was a mass mortality event among great skuas that started in the summer of 2021, with the last HPAIV detection in November 2021 [72,73]. This die-off led to a reduction of 76% of the great skua counts in the UK, as reported by Tremlett *et al.* [74]. The Norwegian isolates originated from birds sampled in the spring and summer of 2022, approximately 1 year after the outbreak in great skuas in the UK from which isolates were obtained (July 2021). This suggests that this particular lineage with genotype C virus has persisted in the bird population, either in great skua or in a prey population common for the skuas and WTEs. The phylogeny of the NA segment was consistent with that of HA, but additional isolates obtained through 2021 from *Anatidae* of the Netherlands (May), *Accipitridae* of Finland (July) and Ireland (November) appeared in very close proximity. This indicates that this lineage of genotype C viruses persisted in Northern Europe during the last half year of 2021 before emerging in Norway during spring.

The other transmission of HPAIV genotype C into Norwegian WTEs clustered with isolates obtained from gulls and swans from Canada and Ireland, predominantly from autumn 2021, and aligns with the transatlantic spread of HPAIV H5N1 virus from Europe to North America in 2021 [75,77]. Subsequent sister lineages of this branch further evolved, leading to massive infections in poultry stocks in North America, followed by the initial introduction of HPAIV H5N1 to South America [78]. The westward transatlantic spread of HPAIV from Europe to North America is well documented [75,77,79]. Pelagic birds, including gulls, migrate between the two continents and converge in the North Atlantic Ocean. This migration pattern raises the possibility of birds transporting novel HPAIV genotypes eastwards from North America to Europe. In our dataset, we found no evidence of eastward transatlantic spread of HPAIV. Further research and additional analyses are necessary to assess this potential transmission pathway, and vigilant surveillance in northern Europe will be crucial for the early detection of any novel viral introductions.

### Detection and persistence of subtype H5N5 (EA-2021-I) in WTEs and other wild birds on mainland Norway

The subtypes H5N1 and H5N5 were detected in WTEs from the west coast and north along the coastline, overlapping with the known geographic distribution of WTEs in Norway. An HPAIV isolate detected in a WTE found in Bodø (Nordland County) in February 2022 represents the index case of subtype H5N5 (genotype I) in Norway. Strikingly, all detections of genotype I in other wild birds on mainland Norway were limited to Finnmark, the northernmost county of Norway. Our phylogenetic analyses of both the HA and NA gene segments show that the H5N5 viruses from WTEs and the other wild birds on mainland Norway cluster tightly together and are most closely related to H5N5 viruses identified in Russia in November 2021 (EPI_ISL_16209277 and EPI_ISL_16209278). The link between the index case of H5N5 in a Norwegian WTE and Russian isolates has been previously reported by Zinyakov *et al.* [82], where they suggest the Caspian Region (Russia) as the possible origin of this H5N5 strain. Transmission into Norway could have occurred via migratory birds with flyways from the region around the Caspian Sea to the northwest and northern parts of Norway. Viruses of genotype I have likely persisted in Norway, as they were detected in both 2022 and late 2023 in WTEs and sporadically detected in other avian species (gulls and other raptors) throughout 2023 and 2024 (Fig. 1b) [64]. In September 2022, HPAIV H5N5 was detected in a sample obtained from a WTE in Finland [83], but we were not able to retrieve the sequences from this isolate and thus could not include it in our phylogenetic analyses. Through 2023 and into 2024, genotype I has reached Iceland, the UK, Germany, Greenland, the Faroe Islands, Canada and Japan [64,84]. We cannot exclude the possibility that some wildlife species may serve as a reservoir for this genotype in Norway. It is possible that certain bird species escape detection in the passive surveillance programme due to subclinical or mild infections caused by genotype I. Such species could be prey for scavenging birds like raptors and gulls, as genotype I has been detected in these bird groups. The sustained presence of genotype I in Norway could also be attributed to favourable conditions for viral longevity, particularly in colder climates. Influenza A virus can retain its infectivity for up to a year in cold and wet environments [85,86].

### The pathological changes in HPAI-infected WTEs were consistent with a multisystemic infection

The clinical signs observed in HPAI-positive WTEs in this study resemble previous observations in WTEs and other raptors infected with HPAIV [11,13,87]. Neurological signs, such as torticollis, ataxia and impaired flight capabilities, indicate dysfunction or damage to the nervous system.

Although gross lesions were absent in all examined WTEs, the PCR Cq values corresponded well with the severity of the histopathological changes as shown by standard light microscopy. In addition, visualization of viral RNA by ISH showed that the positive signal corresponded well with the occurrence of microscopic changes. Cq values were high (34–40) in swab samples from three of the examined WTEs (no. 4, 6 and 12), whereas in eagle no. 8, the Cq value was low (24). Lean *et al.* stated that pathological changes caused by HPAI infection vary between species [88]. This makes the interpretation of macroscopic changes difficult, as there are no pathognomonic changes caused by HPAI infection in birds. Indeed, we encountered the same challenge in this study. It should be pointed out that the changes caused by autolysis and freezing and thawing may have masked some changes and affected the histopathological interpretation. A long, rugged coastline makes finding fresh birds difficult. In addition, our small sample size makes it difficult to draw clear conclusions regarding whether our observations are consistent in WTEs. However, other studies of birds of prey infected with HPAIV, including a study of WTEs, also observe few to no gross lesions in carcasses [12,13, 60, 87, 89].

All four WTEs in this study had histopathological changes in the heart, spleen and brain, but in WTE no. 8, the changes were of a more acute character with widespread necrosis in the heart and spleen. Viral RNA visualization showed a strong signal in the neurons in the brain, confirming a higher viral load in this eagle. A study of bald eagles (*Haliaeetus leucocephalus*), red-tailed hawks (*Buteo jamaicensis*) and great horned owls (*Bubo virginianus*) infected with HPAIV H5N1 displayed consistent histopathological changes in the brain, and a majority of the birds had heart lesions. The condition of other organs displayed more variability [87]. Similarly, the brain and heart lesions were consistently observed in the wild common buzzard (*Buteo buteo*) [60] and WTEs [12] with H5N8 infection, indicating that the brain and heart are common target organs for HPAIVs in raptors. Interestingly, the common buzzards also displayed necrosis of the proventriculus [60], which was not observed in the WTEs in this study or in the other studies of raptors [12,87]. Of note, fibrosis is a pathological process caused by tissue damage. Its chronic nature makes the initial cause difficult to pinpoint, as any inflammatory process can lead to these changes. The absence of viral RNA in these myocardial lesions does not rule out the presence of the virus at an earlier stage of HPAIV infection. Pancreatic necrosis, commonly observed in other birds infected with HPAIV [89], was not observed in any of the WTEs examined in this study. However, given the limited number of individuals studied, it remains uncertain whether WTEs might develop necrosis in this organ at some stage of infection, potentially depending on the virus strain or the progression of the disease.

Lesion profiles may provide an indication of potential infection routes, but it is important to acknowledge that pathology alone is often insufficient to fully determine these routes, as various factors, including the state of carcass preservation, can significantly influence findings. While WTEs ingest HPAIV-infected prey, and infection via the gizzard and intestine could be a plausible route, no pathological changes indicative of this route were observed in the WTEs examined in this study. Another possible route is infection via the olfactory nerve to the brain or haematogenous spread. In one examined WTE (no. 8), viral RNA was detected in the endothelium of vessels in both the brain and heart, suggesting this as a potential infection route. However, a comprehensive understanding of the infection pathways would require a combination of pathology and virology investigations, including *in situ* RNA abundance analysis and viral load assessment by PCR.

## Abbreviations

AIVs: avian influenza viruses
EA: Eurasian
EURL: European Union Reference Laboratory
HA: haemagglutinin
HPAI: highly pathogenic avian influenza
HPAIVs: HPAI viruses
ISH: *in situ* hybridization
LPAIVs: low pathogenic avian influenza viruses
ML: maximum likelihood
MSA: multiple sequence alignment
NA: neuraminidase
NFSA: Norwegian Food Safety Authority
NVI: Norwegian Veterinary Institute
RT-PCR: real-time PCR
WTE: white-tailed eagles
